# A case report of empty Sella syndrome secondary to Hantaan virus infection and review of the literature

**DOI:** 10.1097/MD.0000000000019734

**Published:** 2020-04-03

**Authors:** Haiying Chen, Yuxiang Li, Peng Zhang, Yang Wang

**Affiliations:** a Department of Infectious Diseases, The First Hospital, Jilin University, Changchun, Jilin, China; b Department of Pediatrics, University of Oklahoma Health Sciences Center, Oklahoma City, OK.

**Keywords:** empty sella syndrome, hemorrhagic fever with renal syndrome, imaging examination

## Abstract

**Rationale:**

Bleeding in the anterior pituitary lobe leading to tissue necrosis occurs in the acute stage of severe clinical forms of hemorrhagic fever with renal syndrome (HFRS), while atrophy of the anterior pituitary lobe with diminution of the gland function occurs after the recovery stage. The relationship between Hantaan virus infection and empty Sella syndrome (ESS) has rarely been reported.

**Patient concerns:**

This patient was a 54-year-old previously healthy Chinese male. He presented with fever, headache, and backache with dizziness and oliguria. Physical examination was notable for hypotension and the signs of conjunctival suffusion. His platelets decreased, and his urine protein was positive. Hantaan virus IgM and virus RNA were positive.

**Diagnosis:**

He was diagnosed as having HFRS. In his diuretic phase, his 24-hour urine volume was maintained at 10,000 mL, and his blood pressure was higher for a week. Then, he was diagnosed as having ESS after a series of examinations.

**Interventions:**

Hormone replacement therapy was given to this patient after the diagnosis “ESS” was made.

**Outcomes:**

The patient's symptoms improved, and he was discharged from the hospital soon after hormone replacement therapy.

**Lessons:**

Pituitary function examination and brain magnetic resonance imaging (MRI) need to be considered to scan for ESS and panhypopituitarism in the patients with HFRS accompanied by diabetes insipidus.

## 1 Introduction

The term “empty sella” was first used in 1951 for the neuroradiological or pathological-anatomical exposure of an apparently empty sella turcica. Empty sella syndrome (ESS) is pathophysiologically characterized by either anatomic abnormalities in the diaphragma sellae (primary ESS), damage to the pituitary by irradiation/surgery, or autoimmunity leading to the availability of “empty” space in the sella (secondary ESS).^[1]^ The most prevalent hormone deficiency in this syndrome is growth hormone (GH) deficiency, affecting 35% to 61% of adult patients with ESS.^[2]^ The most common symptom of ESS is headache. Generally, the headache is deep, dull, and centrally situated. Sometimes headache is very severe, along with giddiness and vomiting before imaging examination.^[1]^

In primary ESS, the sella tends to be symmetrically ballooned without evidence of bony lesions. The suprasellar subarachnoid space herniates through an incomplete sellar diaphragm. Cisternography, high resolution computed tomography (CT) scanning, or magnetic resonance imaging (MRI) is used for diagnosis. The signs and symptoms depend upon the size, secretion, and pressure over the pituitary gland. Before the era of CT scan, many cases of empty sella were wrongly diagnosed.^[3]^ Empty sella is the neuroradiological or pathological finding of an apparently empty sella turcica containing no pituitary tissue. The prevalence of primary empty sella, that is, empty sella without any discernible cause, is not precisely known; estimates range from 2% to 20%.^[3]^ Technical advances in neuroradiology have made empty sella an increasingly incidental finding. Improvements in neuroradiological imaging techniques have resulted in an increase in the incidental finding of “empty sella.” According to current data from India, an empty sella turcica without any detectable cause is an incidental finding in approximately 2% of all cerebral MRI scans.^[4]^ In asymptomatic adult patients, the question is whether and to what extent diagnostic hormone testing should be undertaken in an incidental finding of an empty sella, particularly as this does not necessarily have pathological significance.

The causes of most of the primary ESS are still not clear. Hemorrhagic fever with renal syndrome (HFRS) is an acute infective multisystemic disease followed by fever, hemorrhages, and acute renal insufficiency. Bleeding in the anterior pituitary lobe leading to tissue necrosis occurs in the acute stage of severe clinical forms of HFRS, while atrophy of the anterior pituitary lobe with diminution of the gland function occurs after the recovery stage. The relationship between Hantaan virus infection and ESS has rarely been reported, especially in the acute stage.

Herein, we present an adult case with ESS induced by HFRS. He was cured after supportive treatment and hormone replacement therapy.

## 2 Case representation

This patient was a 54-year-old, previously healthy Chinese male with no significant past medical history and surgical history. He lived in a rural area with a high incidence of HFRS. He presented with fever, headache, and backache for 5 days in winter, the epidemic season of HFRS. He suddenly experienced fever up to 40°C. He sometimes felt ocular pain without visual disturbance. He felt dizzy, but he did not have his blood pressure examined. He exhibited oliguria during the last 3 days, but his urine output was not clear. Vomiting and abdominal pain were experienced.

Physical examination was notable for a blood pressure of 88/53 mm Hg, weight = 62 kg, height = 5′7″, and body mass index = 21.5. His body temperature recovered to the normal level of 37°C. Signs of conjunctival suffusion (flush over face, flush over neck, and upper chest) were found.

Laboratory test results were as follows. Routine blood examination showed WBC of 55,000 cells/mL, percentage of neutrophils of 61.9%, and platelets of 93,000 cells/mL. The urine examination showed strong positive protein. Liver enzymes were increased (aspartate aminotransferase 57.3 U/L, alanine aminotransferase 61.0 U/L, gamma-glutamyl transferase 102.0 U/L). Renal function was abnormal (creatinine 138 μmol/L, blood urea nitrogen 7.8 mmol/L). The reverse transcriptase-polymerase chain reaction (RT-PCR) Hantaan virus test result was positive. The Hantaan virus antibody IgM result was positive, while the Hantaan virus antibody IgG was negative. However, the result of antibody IgG to Hantaan virus was positive a week later and the titer of antibody IgG increased 4 times compared with that of the first test. The C-reactive protein (CRP) level was 105 mg/L (0–3.5), and the serum procalcitonin (PCT) was 1.3 μg/L (0.05–0.5).

**Figure 1:**
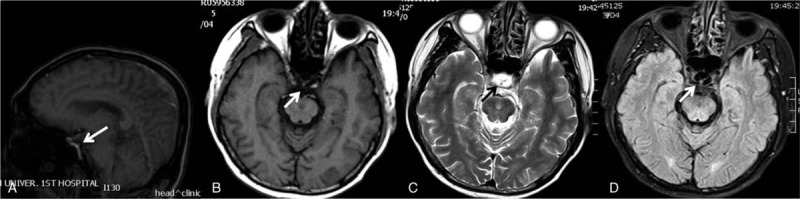
The MRI scan of the brain. (A) Sagittal T1-weighted image to show an empty sella partially filled with cerebrospinal fluid (CSF) (white arrow). (B) T1-weighted image taken in the coronal plane showing hypointensity over the sella region (white arrow). (C) T2-weighted image taken in the coronal plane showing hyperintensity with “black target sign” over the sella region (black arrow). (D) T2-tirm dark fluid image taken in the coronal plane showing hypointensity over the sella region, indicating it is filled with CSF (white arrow).

The urine output during the first 24-hour admission in our department was less than 200 mL. He was diagnosed as overlapping hypotensive phase and oliguric phase of HFRS. After supportive measures treatment, the patient progressed into the diuretic phase within a week. The regular blood cell test result was normal. His urine volume was significantly higher than normal, the 24-hour urine volume was maintained at 10,000 mL, and his blood pressure was obviously higher than his usual level for a week. He felt fatigued and lost his appetite. Then, the examination of pituitary function was performed. All of the results were much lower than the reference range [the urinary free cortisol: 3.95 nmol/24 h (108–961); thyroid-stimulating hormone: 0.075 μIU/mL (0.27–4.2), free T3 1.78 pmol/L (3.1–6.8), free T4 8.2 pmol/L (12–22); 0:00 serum cortisol: <11 nmol/l (240–619), 8:00 serum cortisol: <11 nmol/l (240–619), 16:00 serum cortisol: <11 nmol/l (240–619); 0:00 plasma adrenocorticotropic hormone: 0.22 pmol/L (1.6–13.9), 8:00 plasma adrenocorticotropic hormone: 0.22 pmol/L (1.6–13.9), 16:00 plasma adrenocorticotropic hormone: 0.22 pmol/L (1.6–13.9); prolactinemia: 11.00 mIU/L (55.97–278.36); follicle-stimulating hormone: 0.470 mIU/mL (1.27–19.26); GH: 0.026 ng/mL (0.003–0.971); luteinizing hormone: 0.420 mIU/mL (1.24–8.62); and serum testosterone: <0.35 nmol/L (6.07–27.1)]. The brain MRI result showed that signals of cerebrospinal fluid could be observed in sella (Fig. 1).

The patient's symptoms improved and he was discharged from the hospital soon after hormone replacement therapy (hydrocortisone, L-thyroxine, and testosterone). His urine amount and the results of pituitary function tests recovered to the normal range, and the only discomfort – fatigue – disappeared 1 month later.

## 3 Discussion

HFRS is a zoonotic disease caused by pathogenic hantaviruses. China is the most seriously affected country, with more than 10,000 cases reported annually, and accounts for over 90% of all cases worldwide.^[5,6]^ During 1950 to 2014, the death rate of HFRS was reported as 2.89%, according to the statistical data from the National Health and Family Planning Commission of China.^[7]^

CRP, secreted by the liver in response to bacterial infections, is a parameter used to diagnose infection. PCT, a precursor of calcitonin, is a diagnostic marker of the systemic inflammatory response, with a high sensitivity and specificity for infection. PCT and CRP have been used as new approaches to identify different types of infection.^[8]^ Serum PCT levels are much higher in bacterial, fungal, and parasitic infections than in viral infections or noninfected patients. In epidemic areas, HFRS patients with typical symptoms and routine laboratory findings could soon be diagnosed by experienced professional doctors. However, some cases need a differentiated diagnosis from severe bacterial infection, such as septicemia. The inflammatory biomarkers increased obviously in this case. Elevated levels of CRP and PCT are present in more than 90% of patients with HFRS.^[9]^ The elevated level of PCT in HFRS may be related to immune activation caused by hantavirus.^[10]^ Elevated CRP levels were observed in patients with hantavirus infection. Whether the elevated CRP and PCT are associated with the severity of HFRS remains a controversial issue.^[10–12]^ It is well accepted that the main clinical hallmarks of HFRS are renal failure and hemorrhagic manifestations and that the disease is characterized by laboratory findings indicating renal function impairment, thrombocytopenia, and elevated ALT levels.^[9]^

Vascular endothelial dysfunction is the basic pathological change, and hemorrhage in vital organs could lead to death, especially bleeding in the brain. However, acute impaired pituitary function is very rare in HFRS patients. The main treatment for HFRS is supportive measures. In general, the diuretic phase lasts for approximately 1 to 2 weeks. In this case, the patient's 24-hour urine volume stayed at 10,000 mL, and his fatigue and loss of appetite were obvious. After pituitary function and MRI of the brain were evaluated, impaired pituitary function and empty sella were found. The patient's symptoms, such as polyuria, fatigue, and loss of appetite, were relieved soon after hormone replacement therapy. Secondary empty sella may be caused by pituitary adenomas undergoing spontaneous necrosis (ischemia or hemorrhage). For the most part, pituitary hemorrhage and necrosis were found in cases of death with HFRS via autopsy studies.^[13]^ In a comparative analysis of 897 cases of HFRS, pituitary hemorrhage was revealed at autopsy in 36.8% of the patients, and pituitary necrosis in 5.2%.^[14]^ However, reports about pituitary involvement among survival cases with HFRS are not very common (Table 1). It was regarded as a late sequela in those case reports and was revealed occasionally after many years. Secondary ESS as a complication of HFRS was only reported in 7 cases, among whom ESS was found several years after they were diagnosed as HFRS.^[15,16]^ All 7 cases developed hypopituitarism and were diagnosed by pituitary function and MRI imaging examinations. Their symptoms improved after hormone replacement therapy. “Partly empty ephippium” was revealed as the sequelae of hypophysial hemorrhage in a 54-year-old woman 28 years after she had been diagnosed as HFRS.^[17]^ A case with HFRS accompanied by panhypopituitarism and central diabetes insipidus was recently reported.^[18]^ However, the pituitary MRI findings were normal in that case. In cases with abnormal pituitary function, weakness, apathy, cold intolerance, loss of appetite, hypotension, and loss of libido are the most common symptoms, while only 1 patient had diabetes insipidus. However, our case was diagnosed as ESS in his hospital admission during the diuretic phase of HFRS. Fatigue, loss of appetite, and diabetes insipidus were the major clinical manifestations. His hypertension in this stage might be due to kidney and renin-angiotensin-aldosterone system dysfunction. This is the first acute HFRS case with ESS reported, and there was no pituitary hemorrhage and necrosis found in his MRI result. The pathogenesis of the pituitary injury induced by Hantaan virus infection needs to be determined by further research.

**Table 1:**
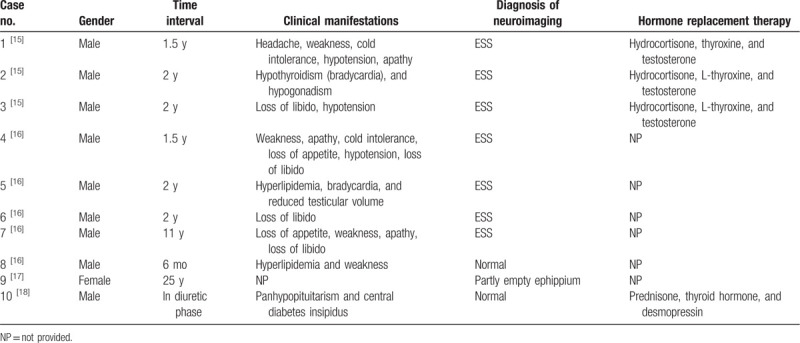
Summary of HFRS cases with abnormal pituitary gland function reported in the literature.

It was recommended that overdiagnosis of empty sella should be avoided, as this might upset patients unnecessarily, and it also incurs substantial expense to the healthcare system.^[19]^ On the contrary, treating hormonal dysregulation has a positive effect on morbidity and quality of life.^[20]^ In the diuretic phase of HFRS, patients need to be monitored carefully. The pituitary function and brain MRI examinations need to be considered to scan ESS and panhypopituitarism in the patients with HFRS accompanied by diabetes insipidus. Appropriate hormone replacement therapy could help the patients achieve relief quickly.

## Acknowledgment

The authors thank the radiological technicians and neurosurgeons for their efforts in the clinical diagnosis and management of this patient.

## Author contributions

**Conceptualization:** Yang Wang.

**Data curation:** Yuxiang Li, Yang Wang.

**Funding acquisition:** Yang Wang.

**Investigation:** Peng Zhang.

**Resources:** Yuxiang Li.

**Software:** Peng Zhang.

**Validation:** Yang Wang.

**Visualization:** Yang Wang.

**Writing – original draft:** Haiying Chen, Peng Zhang.

**Writing – review & editing:** Yang Wang.
